# Induction of labour in mid-trimester pregnancy using double-balloon catheter placement within 12 h versus within 12–24 h

**DOI:** 10.1186/s12884-020-03513-7

**Published:** 2021-01-06

**Authors:** Jing Peng, Ruobing Li, Shuguo Du, Heng Yin, Min Li, Xuan Zheng, Shiyao Wu, Yun Zhao

**Affiliations:** 1 Department of Obstetrics, Maternal and Child Health Hospital of Hubei Province, Tongji Medical College, Huazhong University of Science and Technology, No. 745, Wuluo Road, Hongshan District, Wuhan, 430070 China; 2 Department of Gynaecology and Obstetrics, Wuhan University of Science and Technology, No.2, Huangjiahu West Road, Hongshan District, Wuhan, 430065 China

**Keywords:** Cervical ripening, Double balloon catheter, Mid-trimester, Pregnancy termination, Induced abortion

## Abstract

**Background:**

This study aims to evaluate the efficacy and safety of the induction of labour in mid-trimester pregnancy using a double-balloon catheter (DBC) within 12 h versus within 12–24 h.

**Methods:**

In this retrospective study, a total of 58 pregnant women at 14 + 0 weeks to 27 + 6 weeks of gestation were enrolled as research subjects, and they underwent the intended termination of pregnancy at our birth centre from January 1, 2017, to June 31, 2019. Based on the duration of DBC, the patients were divided into two groups, namely, the DBC group within 12 h and the DBC group within 12–24 h.

**Results:**

All 58 cases were successful vaginal deliveries, and no one chose to undergo caesarean section. The success rate of induction (successful abortion of the foetus and placenta without the implementation of dilation and evacuation) was higher in the DBC group within 12–24 h (96.3%, 29/31) than in the DBC group within 12 h (71.0%, 18/27) (*p* < 0.05). Additionally, the time from DBC removal to delivery in the DBC group within 12–24 h was significantly shorter than that in the DBC group within 12 h (3.0 h versus 17.8 h) (*p* < 0.05), and the degree of cervical dilation after DBC removal in the DBC group within 12–24 h was larger than that in the DBC group within 12 h (*p* < 0.05).

**Conclusion:**

In the clinic, the placement time of DBC generally lasts for approximately 12 h. However, considering that the cervical condition is immature in the mid-trimester, properly extending the placement time of DBC to 24 h will benefit cervical ripening and reduce the chance of dilation and evacuation.

## Background

In prenatal screening, we often use ultrasound to measure the thickness of the foetal nuchal translucency at 11 + 0 to 13 + 6 weeks of gestation and carry out maternal serum screening to screen for foetal aneuploidy chromosome abnormalities in early pregnancy. In addition, ultrasound examination is normally performed at 20 + 0 to 24 + 6 weeks of gestation to screen for structural abnormalities [1]. After learning about severe foetal abnormalities and their poor prognosis, most families choose to terminate pregnancy mid-trimester [2]. Induction of labour is a common obstetric intervention that occurs in a high proportion of pregnancies [3]. Both medical and surgical methods are available for mid-trimester pregnancy. Dilation and evacuation procedures (D&E) are more common methods in the United States. In contrast, medical methods such as mifepristone plus misoprostol are more common in the United Kingdom, Europe and developing countries [4, 5]. In our country, the common approach for the induction of labour in mid-trimester pregnancy is to use pharmacological and mechanical devices. Pharmacological devices include mifepristone combined with ethacridine lactate or mifepristone combined with misoprostol [6]. The mechanical devices include transcervical Foley balloon catheters and cervical double balloon catheters (DBCs). Single balloon and DBC have been increasingly used in recent years at term with an immature cervix [7, 8] or for pregnant women with a history of previous caesarean section [3]. Mechanical methods are the earliest approaches to develop a mature cervix, and their effectiveness level is equivalent to that of prostaglandins [9, 10]. Balloon treatment is well accepted by pregnant women [11, 12] and will not cause excessive stimulation or poor foetal heart monitoring changes [13].

In our medical centre, the common method for termination of mid-trimester pregnancy is to apply mifepristone combined with ethacridine lactate or misoprostol. We have also applied mifepristone combined with DBC for cases with liver and renal dysfunction, oligohydramnios and failure of cervical ripening after using ethacridine lactate or misoprostol.

In this retrospective study, we evaluated the efficacy of DBC within 12–24 h versus DBC within 12 h on the termination of mid-trimester pregnancy from January 1, 2017, to June 31, 2019 in our birth centre.

## Methods

### Ethical approval and patient consent

The study protocol was approved by the Ethics Committee of Maternal and Child Health Hospital of Hubei Province ([2019] IEC (XM008)). All included women signed written informed consent for therapeutic procedures and for the publication of those reports.

### Selection of patients and study design

**Fig. 1:**
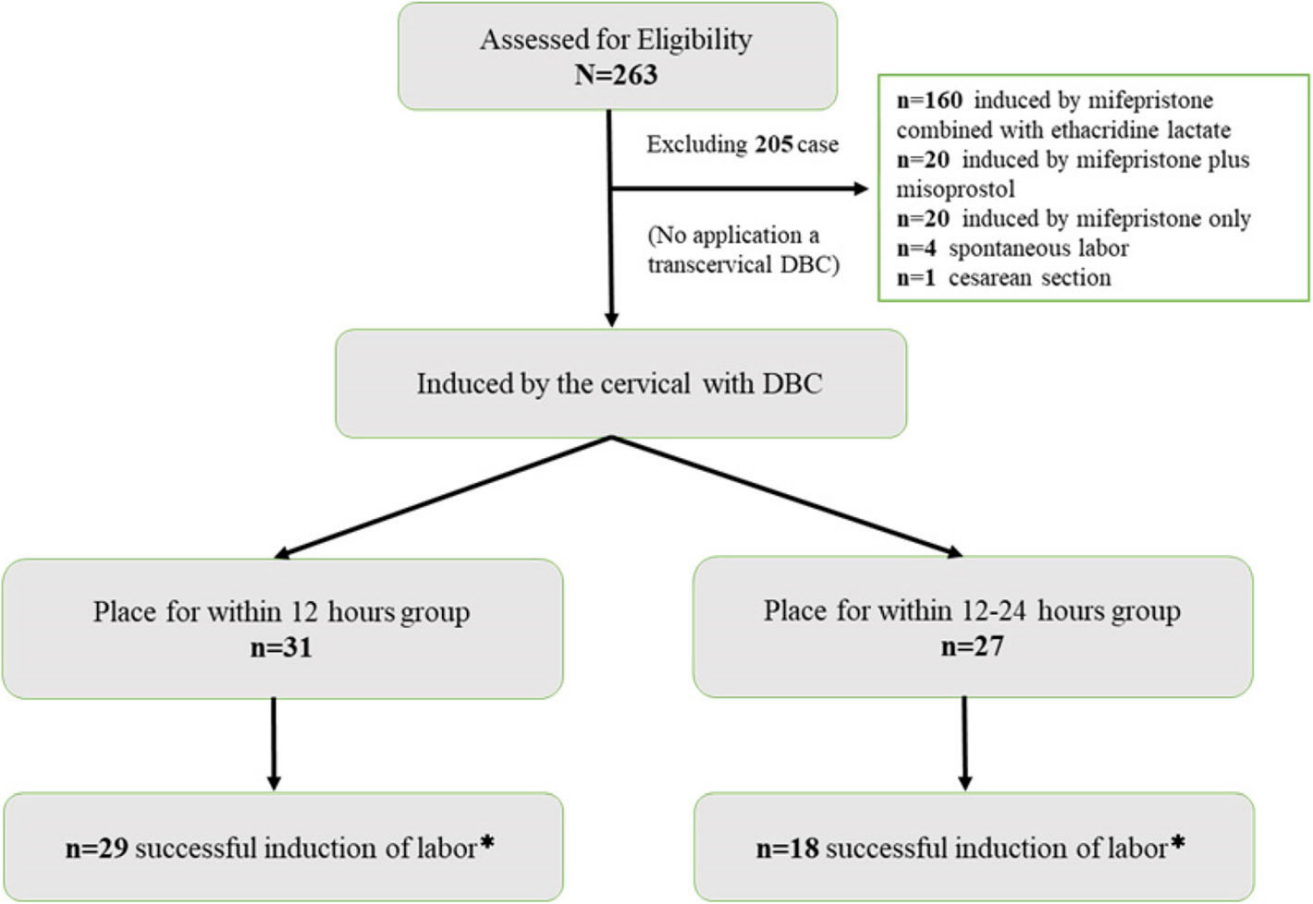
Flowchart demonstrating. *successful abortion of fetus and placenta without dilatation and evacuation

The flowchart of the experimental programme design is shown in Fig. 1. In this retrospective study, we included pregnant women with gestational ages between 14 + 0 weeks and 27 + 6 weeks. All included women underwent intended termination of pregnancy at our birth centre from January 1, 2017, to June 31, 2019. Our centre is a large birth centre in China. During the observation period, 67,540 new babies were born in our centre. Regarding the inclusion criteria, pregnant women aged 18 to 50 years who underwent DBC to induce labour for foetal death, foetal anomalies and serious maternal complications that prevented continuous pregnancy were included in this study. For exclusion criteria, those aged less than 18 years or older than 50 years and those who did not undergo DBC to induce labour (such as mifepristone combined with ethacridine lactate, mifepristone plus misoprostol or caesarean section) were excluded from this study. A total of 263 patients (the incidence of labour induction for congenital anomalies, foetal death or severe maternal complications in the mid-trimester was 3.9% (263/67540)) were selected in this study, including 10 cases using uterine artery embolization (UAE) for prenatal haemorrhage and 1 case for postpartum haemorrhage. The rate of UAE was 4.2% (11/263). Among them, 160 cases undergoing induction of labour by using mifepristone combined with ethacridine lactate, 20 cases undergoing induction of labour via mifepristone plus misoprostol, 20 cases undergoing induction of labour by mifepristone only, 4 cases of spontaneous labour and 1 case of caesarean section were excluded from this study. The remaining 58 cases were included in our study. Based on the indwelling time of DBC, the 58 cases were divided into two groups: group 1 with DBC within 12 h (0<DBC time ≤ 12 h) containing 31 cases (the 12 h group), and group 2 with DBC within 12–24 h (12<DBC time ≤ 24 h) containing 18 cases (the 24 h group).

### DBC [2]

DBC is usually used according to the manufacturer’s instructions (Cervical Ripening Balloon; Cook OB/GYN, Spencer, IN, USA). It involves 2 balloons (uterine and vaginal balloons), and each balloon can fill with a maximum of 80 mL of normal saline. First, the uterine balloon (red piston, marked with “U”) is placed into the lower part of the uterine cavity, and 40 mL of normal saline solution is injected into it. Then, the vaginal balloon (green piston, marked with “V”) is placed outside the cervical orifice, and 40 mL of normal saline solution is injected into it. After vaginal examination to check if DBC is placed normally, the fluid amount in both balloons is alternatively increased by 20 mL each time until each balloon reaches 80 mL. After ensuring that the balloons are positioned correctly, the proximal end of the catheter is fixed to the inside of the patient’s thigh. If the patient feels that symptoms of sweating or flustering are unbearable, then 10–20 mL of normal saline is withdrawn from both balloons until the patient can tolerate the DBC.

Based on the judgement of the attending physician on the cervical condition of the pregnant women in mid-trimester pregnancy, the longest placement time of DBC is set to 12 h or 24 h, but the device should be removed immediately upon the occurrence of any of the following events, including spontaneous labour, expulsion, spontaneous ruptured membranes, or unexplained vaginal bleeding [14]. If those events do not happen, the DBC device will be removed after holding for 12 h in the DBC group within 12 h and for 24 h in the DBC group within 12–24 h.

### Intervention for pregnancy termination

In our hospital, DBC is not the first option for termination of pregnancy mid-trimester. We normally use DBC in the two following situations. First, it is used for difficult induction of labour. Oral mifepristone combined with extra-amniotic administration of ethacridine lactate (Rivanol) is applied after admission, 150 mg of mifepristone is administered to the patient for 3 days (each pill contains 25 mg of mifepristone, 2 pills a day), and an injection of 100 mg of ethacridine lactate is performed at approximately 9:00 AM on the 4th day. Then, patients can normally be discharged in the following 24–48 h. If the patients still exhibit no response and their cervixes are immature in the following 72 h, then DBC will be used. Second, for patients with liver and/or renal dysfunction and/or oligohydramnios, we use mifepristone plus DBC directly. Mifepristone (150 mg) is administered to the patient for 3 days, and DBC is used at approximately 9:00 AM on the 4th day. After removing the DBC, oxytocin is infused for both groups to assist labour at a dose of 2.5–5.0 units in 500 mL Ringer’s solution, with an infusion rate of 8–40 mL/h. At the same time, surgical methods of dilation and evacuation are used by experienced obstetricians when the body temperature of cases reaches 38.5 °C or massive antenatal haemorrhage occurs.

### Observation indicators

Gestational age was estimated based on ultrasonography performed at 11 + 0 to 13 + 6 weeks. The Bishop scoring system is based on a digital cervical exam of a patient, with zero points as the minimum and 13 points as the maximum. The scoring system can be used to evaluate cervical dilation, position, effacement, consistency of the cervix, and foetal station. Cervical dilation, effacement, and foetal station are allocated 0 to 3 points, while cervical position and consistency are given 0 to 2 points [1]. To compare the efficacy of DBC in the two groups, the primary outcome is the success rate of labour induction, which means successful abortion of the foetus and placenta without implementation of dilation and evacuation. The secondary outcomes include the time from induction of DBC to labour and the time from removing the DBC to delivery, as well as parameters of maternal and foetal outcomes, such as the rate of antepartum haemorrhage, UAE before or after delivery, postpartum haemorrhage (PPH), and puerperal infection.

### Statistical methods

All analyses were conducted using the Statistical Package of Social Sciences software (SPSS Version 13.0 Inc., Chicago, IL, USA). The values and variables are reported as the means±standard deviation. Student’s t-test was performed to compare the variables in a Gaussian distribution. The chi-square test was used to evaluate the categorical variables. The Wilcoxon test was used to evaluate the difference in a non-Gaussian distribution between the two groups. The difference was considered statistically significant at *p* < 0.05.

## Results

### Demographic data of the 12-h group and the 24-h group

The baseline data and pregnancy characteristics of the two groups are listed in Table 1. There were no significant differences in maternal age, gestation, parity, nulliparity, maternity insurance, gestational age at termination, placenta previa rate, history of previous caesarean section, or body mass index between the two groups (*p* > 0.05). There was no significant difference in reasons for pregnancy termination between the two groups (*p* > 0.05).

**Table 1:** Baseline demographics and pregnancy characteristics

Groups	12 h-CRB (*n* = 31)	24 h-CRB (*n* = 27)	t/Z/X^2^	*P*
Age(y, X̅ ± s)	28.6 ± 5.5	31.2 ± 4.5	−1.969	0.054
Gestation (n, min-mix)	1–6	1–6	−1.890	0.059
Parity (n, min-mix)	0–2	0–3	−0.679	0.497
Nulliparous (n, %)	4, 12.9	3, 11.1	1.000	0.579
Maternity insurance (n, %)	12, 38.7	7, 25.9	1.071	0.301
Gestational age (wk, X̅ ± s)	23.0 ± 6.1	22.2 ± 6.2	0.528	0.600
History of previous cesarean delivery (n, %)	8, 25.8	11, 40.7	1.461	0.227
Body mass index (kg/m^2^, X̅ ± s)	22.7 ± 3.4	23.7 ± 4.6	−0.924	0.359
Placenta previa (n, %)	6, 19.4	9, 33.3	1.471	0.225
Indications for pregnancy termination				
Fetal death	7	8		
Fetal anomaly	23	18	−0.530	0.596
Severe complications	1	1		
Methods of induce (n,%)				
Failure of ethacridine	15 (48.4)	7 (25.9)	3.092	0.079
Mifepristone + DBC	16 (51.6)	20 (74.1)		

Student’s test, Chi- square test and Wilcoxon test were used

### Cervical ripening before and after DBC in the 12-h group and in the 24-h group

There was no significant difference in cervical ripening between the two groups according to Bishop scores (*p* > 0.05). However, after ripening by DBC, the cervix ripened more in the 24 h DBC group than in the 12 h DBC group (*p* < 0.05) (Tables 2 and 3).

**Table 2:** The Bishop scores of cervices before DBC insert between in the two groups

	n	0	1	2	≥3
12 h DBC	31	0	12	10	9
12-24 h DBC	27	0	5	15	7
Z	0.867				
*P*	0.386				

Wilcoxon test was used

**Table 3:** The dilation of cervix after taking out DBC between in the two groups

	n	0(cm)	1(cm)	2(cm)	≥3(cm)
12 h-DBC	31	10	9	10	2
24 h-DBC	27	3	9	7	8
Z	−2.185				
*P*	0.029				

Wilcoxon test was used

### The time from induction and DBC removal to delivery

The time from induction to delivery in the 24-h group was shorter than that in the 12-h group (median time, 27.0 h versus 29.8 h), but the difference was not statistically significant (*p* > 0.05). However, the time from DBC removal to delivery in the 12-h group (median time, 17.8 h) was longer than that in the 24-h group (median time, 3.0 h), indicating a significant difference (*p* < 0.05) (Table 4).

**Table 4:** The time from induction and DBC removal to delivery

Lasting time	12 h-DBC (*n* = 31)	12-24 h DBC (*n* = 27)	Z	*P*
Induction to delivery (h, Median,95%CI)	29.8 (19.0–35.7)	27.0 (24.7–30.2)	−0.405	0.685
DBC removal to delivery (h, Median,95%CI)	17.8 (7.0–23.7)	3.0 (0.7–6.2)	−4.366	0.000

Wilcoxon W test was used

### The maternal and foetal outcome parameters in the two groups

Within 12–24 h, 2 patients (3.7%, 2/27) underwent dilation and evacuation after removing DBC, while 9 patients in the within 12 h DBC group underwent dilation and evacuation (29.0%, 9/31) (*p* < 0.05) (Table 5). None of the groups underwent caesarean section to induce labour, and all of them had successful vaginal delivery.

**Table 5:** The maternal and fetal outcome parameters between in the two groups

Groups	12 h-CRB (*n* = 31)	24 h-CRB (*n* = 27)	t/Z/X^2^	*P*
ICU (n, %)	5, 16.1	4, 12.9	0.019	1.000
Puerperal infection (n, %)	3, 9.7	5, 18.5	0.949	0.453
Antepartum hemorrhage (n, %)	5, 16.1	2, 7.4	1.034	0.432
UAE before delivery (n, %)	4, 12.9	2, 7.4	0.470	0.675
Dilatation and evacuation	9, 29.0	2, 3.7	4.391	0.047
Weight of fetus (g, Median, 95%CI)	340 20–1000	300 45–1045	−0.047	0.963
Blood loss at delivery				
Volume (mL, X̅ ± s)	271.4 ± 131.6	306.2 ± 89.6	−1.161	0.251
PPH>500 ml (n, %)	5, 16.1	3, 11.1	0.306	0.712

T test, Wilcoxon W test, Chi-square test and Fisher exact test are used

*UAE* Uterine artery embolization

There were no significant differences in the weight of the foetus, blood loss at delivery, rate of antepartum haemorrhage, rate of puerperal infection, UAE before delivery, rate of PPH, or rate of intensive care unit (ICU) between the two groups (*p* > 0.05) (Table 5). Neither group used UAE for postpartum haemorrhage.

### The WBC count and haemoglobin level in the two groups

There was no significant difference in the WBC cell count or haemoglobin at admission and discharge between the two groups (*p* > 0.05) (Table 6).

**Table 6:** The WBC cell count and hemoglobin in the two groups

Groups	12 h-CRB (*n* = 31)	24 h-CRB (*n* = 27)	t	*P*
WBC at admission (10^9^, X̅ ± s)	8.9 ± 2.3	9.4 ± 2.1	−0.720	0.474
WBC at discharge (10^9^, X̅ ± s)	13.3 ± 3.5	13.0 ± 2.0	0.364	0.717
Hb at admission (g/L, X̅ ± s)	115.4 ± 11.8	113.9 ± 11.2	0.507	0.614
Hb at discharge (g/L, X̅ ± s)	108.3 ± 11.3	103.8 ± 12.0	1.467	0.148

T test was used

### Hospitalization days and expenditure

In the 24-h group, the average hospitalization duration was 9.8 d, which was shorter than that in the 12-h group (12.3 d) (*p* < 0.05). At the same time, the hospitalization expenditure in the 24-h group was lower than that in the 12-h group (*p* < 0.05) (Table 7).

**Table 7:** Hospitalization days and expenditure of inpatients

Groups	12 h-CRB (*n* = 31)	24 h-CRB (*n* = 27)	t/Z/X^2^	*P*
Hospitalization days (d, X̅ ± s)	12.3 ± 4.4	9.8 ± 3.8	2.258	0.028
Expenditure of Inpatients (RMB, Median, 95%CI)	13,810.5 3423.6–32,659.9	5217.9 3056.7–34,215	−2.019	0.044

T test, Wilcoxon W test, Chi-square test, Fisher exact test

## Discussion

In recent years, the former one-child policy has been gradually replaced by the universal two-child policy (2015). With the increasing rate of multiparous women in China, birth defects are a challenging issue. Women at very advanced maternal age (≥43 years) have a higher risk of preeclampsia, intrauterine growth retardation, stillbirth, and placental abruption than their younger counterparts [15]. Zhang X et al. [16] analysed 1,260,684 births from the surveillance system in Zhejiang Province, China, and found that the rates of birth defects during 2013, 2015, and 2017 were 245.95, 264.86, and 304.36 per 10,000 births, respectively, and there were age-related anomalies after the release of China’s new two-child policy. There are many clinical problems during induced labour that need to be solved, especially complete placenta previa [17] and immature cervical conditions [2]. An increasing number of women in developing countries choose to postpone pregnancy [18], and studies have found an association between older pregnant women and a high risk of chromosomal abnormalities, miscarriages and preterm birth with gestational ages of less than 34 weeks. In addition, stillbirths are more common in women aged 35–39 [18]. Therefore, the methods, safety, effectiveness and postoperative complications of labour induction in mid-trimester pregnancy are worth pondering and exploring. Our study observed pregnant women who underwent the intended termination of pregnancy for foetal death, foetal anomalies and serious maternal complications in mid-trimester pregnancy. Of those 58 selected cases, there were 22 cases using DBC for lasting immature cervical condition after applying mifepristone combined with rivanol or mifepristone plus misoprostol, and the other 36 cases were subjected to mifepristone plus DBC directly for liver and kidney dysfunction or oligohydramnios.

The mechanism of DBC-based labour induction is basically the compression effect of the DBC balloon on the cervix, which leads to the release of endogenous prostaglandins [10]. In addition to the local effect, mechanisms that involve neuroendocrine reflexes (such as the Ferguson reflex) may promote the onset of contractions [9]. Researchers have evaluated the effectiveness of these devices by comparing them with prostaglandins and have reported that they are equally effective, and the incidence of tachycardia is lower than that of prostaglandins [10]. Placing a transcervical DBC can be the primary method or one of the alternative medical methods if the patient and/or obstetrician prefers not to conduct surgical operation [2]. DBC is commonly used for cervical ripening to induce labour with and without prior caesarean section in term [19, 20]. Korb D et al. [19] compared the effectiveness of cervical ripening by DBC (*n* = 117) and prostaglandins (*n* = 127) in women with a previous history of caesarean delivery and an unfavourable cervix (Bishop score<6) and found no significant difference between them in terms of caesarean rate and the median interval between the start of ripening and delivery (42.5% and 28.7 h in the prostaglandin group vs 42.7% and 25.6 h in the DBC group). There have been very few studies on induced labour in mid-trimester pregnancy using DBC.

In the clinic, the placement time of DBC generally lasts for 12 h. In our experiment, the placement time of DBC was extended to 24 h for the first time if there was no occurrence of spontaneous labour, expulsion, or spontaneous rupture of membranes. We compared the effects of DBC within 12 h and within 12–24 h for the induction of labour in mid-trimester pregnancy. We found that the success rate of induction of labour was higher in the DBC group within 12–24 h (96.3%, 29/31) than in the DBC group within 12 h (71.0%, 18/27). It is known that properly extending the time of DBC can reduce the chance of surgical induction of labour, thereby reducing maternal damage and helping to obtain complete foetal tissues. Although there was no significant difference in the time from induction to delivery between the two groups, the time from DBC removal to delivery in the 24-h group was significantly shorter than that in the 12-h group (3.0 h versus 17.8 h). This may help reduce the risk of fever and labour pain by using pharmacological methods to assist labour. In addition, the hospitalization days and expenditures in the 24-h group were lower than those within 12 h. There were no significant differences in the rate of antepartum haemorrhage, rate of UAE before delivery, rate of PPH, rate of ICU care or in the WBC cell count and haemoglobin at admission and discharge between the two groups, but the hospitalization days were longer and expenditure was higher in the 12-h group.

## Conclusion

Clinically, the placement time of DBC generally lasts for approximately 12 h, and the cervical condition is still immature after removal of DBC in mid-trimester pregnancy. Properly extending the placement time of DBC to 24 h can be beneficial for cervical ripening and reducing the chance of dilatation and evacuation in mid-trimester pregnancy.

### Limitations

First, this study was a retrospective study in which the data were only collected from patients’ medical records, and the Bishop score was the only index for cervical evaluation.

Second, the most serious complication for induced labour in mid-trimester pregnancy by DBC lasting for 12 h to 24 h was infection; therefore, sensitive indices of infection should be added in addition to body temperature and WBC count.

Last, there may be acknowledged possible selection bias as patients were allocated to each group according to baseline cervical condition.

## Data Availability

Access to the qualitative data will be given upon request to the corresponding author after taking any necessary precautions to safeguard participants’ privacy and confidentiality.

## Abbreviations

DBC: Double-balloon catheter
D&E: Dilation and evacuation procedures
UAE: Uterine artery embolism
PPH: Postpartum haemorrhage
ICU: Intensive Care Unit
